# Alternative Splicing of Homologous Recombination Genes Is Associated with Homologous Recombination Deficiency and Immunoregulatory Phenotypes in Breast Cancer

**DOI:** 10.3390/biomedicines14102231

**Published:** 2026-10-02

**Authors:** Ahmed M. Basudan, Yazeed Alshuweishi, Hamood AlSudais, Mohammad A. Alfhili

**Affiliations:** Department of Clinical Laboratory Sciences, College of Applied Medical Sciences, King Saud University, Riyadh 12372, Saudi Arabiahalsudais@ksu.edu.sa (H.A.); malfeehily@ksu.edu.sa (M.A.A.)

**Keywords:** alternative splicing, homologous recombination deficiency, breast cancer, *RAD54B*, immunoregulatory phenotypes

## Abstract

**Background/Objectives:** Homologous recombination deficiency (HRD) is an important feature of breast cancer, but the relationship between alternative splicing (AS) of homologous recombination (HR) genes and HRD remains poorly characterized. The study investigated whether coordinated HR-gene AS is associated with genomic HRD and immune phenotypes. **Methods:** Using The Cancer Genome Atlas Breast Invasive Carcinoma (TCGA-BRCA) cohort, we developed a normal-referenced, module-balanced HR splicing disruption score (HRSD) from 186 AS events across 49 HR genes. Associations with HRD were evaluated across molecularly restricted subsets and by multivariable analysis. HRD-associated splicing features were assessed across six immune phenotypes. A prioritized *RAD54B* alternative termination event was characterized further and independently evaluated in the Sweden Cancerome Analysis Network-Breast (SCAN-B) cohort. **Results:** HRSD was positively associated with genomic HRD (*ρ* = 0.338, *p* = 1.64 × 10^−21^) and remained significant after restriction for HRD-associated molecular features and exclusion of basal tumors. In multivariable models, each 1-SD increase in HRSD was independently associated with higher HRD scores (β = 3.17, *p* = 7.08 × 10^−7^) and HRD-high status (OR = 1.6, *p* = 0.001). HRD-associated splicing converged with immunoregulatory phenotypes, particularly regulatory T-cells and checkpoint activity. *RAD54B* alternative termination (RAD54B|AT|84553) was higher in HRD-high tumors (*p* = 3.42 × 10^−14^) and remained independently associated with immunoregulatory phenotypes after adjustment. SCAN-B analysis validated the terminal-exon structure and reproduced subtype-specific termination patterns. **Conclusions:** HR-associated AS represents a transcript-level dimension of HRD biology in breast cancer. *RAD54B* alternative termination may provide a candidate link between HRD-associated splicing and immunoregulatory phenotypes, warranting further functional investigation.

## 1. Introduction

Homologous recombination (HR) is a high-fidelity DNA repair pathway that plays a central role in the repair of DNA double-strand breaks and the maintenance of genomic stability [1,2]. Deficiency in HR repair results in characteristic patterns of genomic instability, including chromosomal alterations and genomic scars that can be used to identify tumors with homologous recombination deficiency (HRD) [2,3]. In breast cancer, HRD is associated with distinct tumor biology that carries important therapeutic implications. Notably, the deficiency increases sensitivity to DNA-damage agents and poly(ADP-ribose) polymerase (PARP) inhibitors [2,4]. Although pathogenic alterations in *BRCA1* and *BRCA2* are among the best-established causes of HRD, disruption of other components of the HR machinery can also impair HR repair [5].

The HRD phenotype is not restricted to tumors harboring *BRCA1/2* mutations. Alterations in other HR-related genes and epigenetic silencing of *BRCA1* through promoter methylation can also contribute to HR dysfunction [5,6]. Moreover, genomic and mutational signature approaches have identified HRD-like features in *BRCA1/2* wild-type breast cancers, indicating that genomic evidence of HR dysfunction extends beyond tumors carrying canonical *BRCA1/2* alterations [7,8]. More broadly, the molecular heterogeneity of breast cancer has motivated the identification of subtype- and context-specific molecular biomarkers [9]. While comprehensive molecular profiling revealed many genetic and epigenetic mechanisms in HRD, genomic HRD phenotypes are not always attributable to *BRCA1/2* mutation status alone [10]. These observations support the investigation of additional molecular features associated with HR dysfunction in breast cancer.

Alternative splicing (AS) represents a potential additional layer of molecular variation affecting gene and protein function. By generating distinct transcript isoforms, AS contributes to transcriptomic and proteomic diversity, while dysregulated splicing can alter protein function and has been implicated in multiple aspects of cancer biology [11,12]. RNA processing and DNA-damage response are closely interconnected, with DNA damage influencing the splicing of genes involved in DNA repair, cell-cycle regulation, and apoptosis [13]. Alternative transcript isoforms have been described for key HR genes, including *BRCA1* and *BRCA2*, providing evidence that components of the HR machinery can undergo substantial transcript-level variation [14,15]. More recently, altered splicing of multiple DNA-repair genes has also been demonstrated in breast cancer models [16]. Despite these observations, the relationship between coordinated AS across the HR machinery and genomic HRD in breast cancer remains poorly characterized.

HRD may also influence the tumor immune microenvironment. Genomic instability resulting from defective DNA repair can affect tumor immunogenicity and immune signaling. However, the relationship between HRD and anti-tumor immunity is complex and does not necessarily reflect uniform immune activation [17]. In breast cancer, genomic features of *BRCA* deficiency and HRD have been associated with variation in immune-related measures, including cytolytic activity, immune infiltration, and interferon-related signatures [17]. At the same time, regulatory T-cells and immune-checkpoint pathways can contribute to an immunoregulatory tumor microenvironment and suppression of effective anti-tumor responses [18,19]. However, whether splicing of HR genes is associated with immunoregulatory phenotypes in breast cancer remains insufficiently understood.

In this study, we investigated whether HR-gene AS is associated with genomic HRD and immune phenotypes in breast cancer. Using TCGA-BRCA, we developed a normal-referenced, module-balanced HR disruption framework, assessed its relationship with HRD across molecular contexts, and evaluated HRD-associated splicing features across immune phenotypes. A prioritized *RAD54B* alternative termination event was further characterized and independently evaluated in the SCAN-B cohort.

## 2. Materials and Methods

### 2.1. Study Design and Data Sources

**Figure 1:**
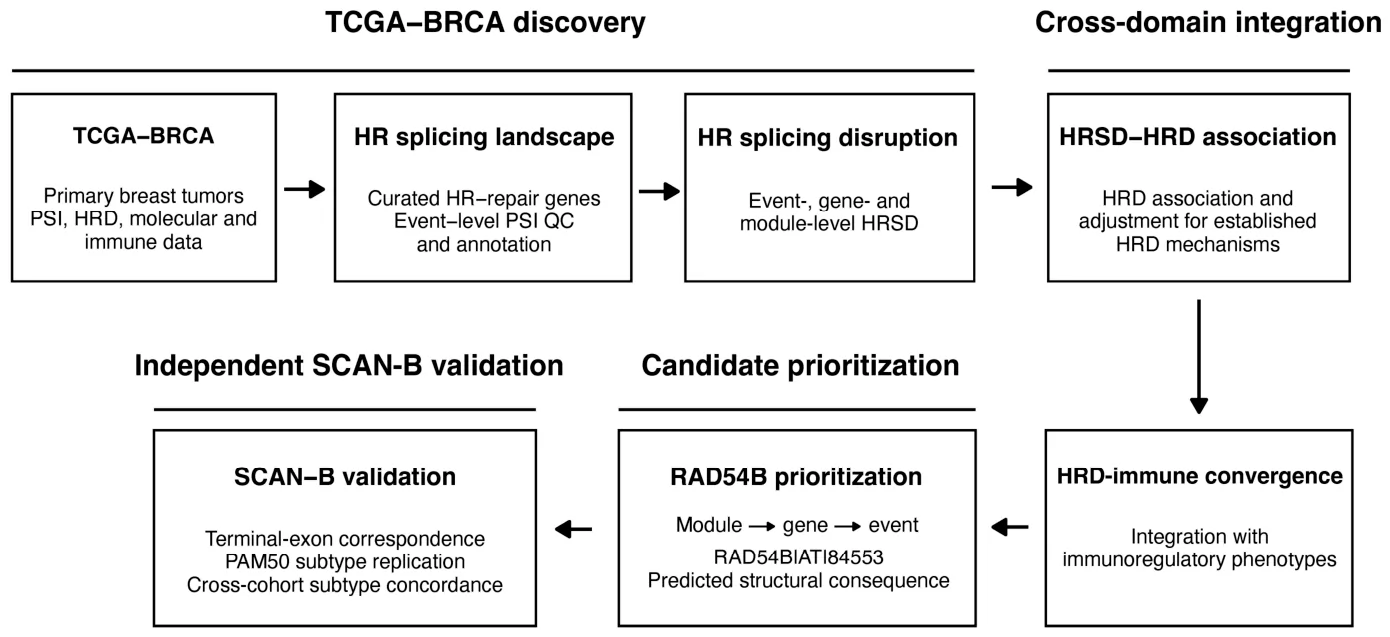
Study design and analytical framework. TCGA-BRCA was used to characterize alternative splicing (AS) of homologous recombination (HR) genes and construct the HR splicing disruption score (HRSD). Associations with homologous recombination deficiency (HRD) and immune phenotypes were evaluated, leading to cross-domain prioritization and characterization of *RAD54B* alternative termination. Independent validation in SCAN-B assessed terminal-exon structure and PAM50 subtype-associated patterns. PSI, percent spliced in; PAM50, Prediction Analysis of Microarray 50.

The study used an integrative framework to investigate the relationship between alternative splicing (AS) of homologous recombination (HR) repair genes, homologous recombination deficiency (HRD), and immunophenotypes in breast cancer (Figure 1). The Cancer Genome Atlas Breast Invasive Carcinoma (TCGA-BRCA) cohort was used as the primary discovery cohort. Alternative splicing profiles were downloaded directly from “TCGASpliceSeq” [20] database as event-level Percent Spliced In (PSI) matrices. Primary breast tumors were used for the principal tumor-based analyses, while normal-adjacent breast tissue was retained for tumor vs. normal comparisons of prioritized splicing events. PSI data were subsequently processed and quality-controlled as described in Section 2.3. PAM50 molecular subtype annotations for TCGA-BRCA were obtained from the National Cancer Institute (NCI) Genomic Data Commons (GDC) TCGA-BRCA publication resource [21,22].

Genomic HRD data were downloaded as reformatted scores from the “TCGAhrd” repository [23], originally derived from the NCI-GDC DNA damage repair (DDR) resource [24] and previously described by Knijnenburg et al. [25]. Somatic mutation data for TCGA-BRCA were retrieved using the “TCGAbiolinks” package in R [26,27]. Open-access masked somatic mutation data generated by the Aliquot Ensemble Somatic Variant Merging and Masking workflow were used to determine *BRCA1/2*, *TP53*, and additional HR-gene somatic alteration status. DNA methylation data were obtained from the NCI-GDC TCGA-BRCA 2012 publication resource [21,22]. The combined HumanMethylation27 and HumanMethylation450 array dataset was used to derive *BRCA1* promoter methylation status. Methylation values from four promoter-associated CpG probes were summarized. Immune-related data were obtained from the NCI-GDC PanCancer Atlas publication resource [28,29], including immune gene-signature scores, leukocyte fraction estimates, and CIBERSORT-derived immune cell fractions [30]. These measures were processed into immunophenotypes.

For external validation, transcript-level expression data and corresponding transcript annotations for the independent SCAN-B breast cancer cohort (GSE96058) were obtained from the NCBI Gene Expression Omnibus (GEO) repository [31]. Sample phenotype and PAM50 subtype annotations were retrieved from the GEO series matrix using the “GEOquery” R package [32].

### 2.2. Definition of the Homologous Recombination Gene Set and Functional Modules

A curated HR gene set was constructed from human HR-related pathway annotations obtained from the Molecular Signatures Database (MSigDB) version 25.1.1 [33] using the “msigdbr” (version 2.76.0) R package [34]. Genes represented in at least two prespecified HR-related gene sets were retained. To ensure representation of established components of the core HR machinery that might not meet this pathway overlap criterion, a predefined set of mechanistically established HR genes was additionally retained based on established HR biology [35,36,37,38,39]. The union of these criteria generated a 73-gene HR set (Supplementary Table S1).

Genes were assigned to functional modules according to their established roles in HR and associated DNA-damage response. The classification was designed to represent major functional components of the HR process. Eight modules were defined for the primary HR splicing disruption framework: BRCA-PALB2 coordination, damage recognition/end resection, Fanconi HR interface, HR damage signaling, RAD51 loading/strand invasion, recombination intermediate processing/resolution, repair DNA synthesis, and single-stranded DNA protection. POLH and POLK were classified separately as translesion-synthesis genes and were excluded from the primary module-based HR splicing disruption framework (Supplementary Table S1).

### 2.3. Alternative Splicing (AS) Data Processing and Quality Control (QC)

Samples were classified using TCGA identifiers, and PSI matrices were separated into primary tumor and normal breast tissue samples. The normal-reference pool comprised 113 normal-adjacent breast tissue samples. Event-level QC required PSI measurements in at least 70% of primary tumors and at least five normal breast samples. Because splicing disruption was subsequently standardized relative to normal breast tissue, events were additionally required to have a measurable normal-tissue dispersion. Because PSI availability varied across events, the normal reference was calculated separately for each event using the available normal samples. Among the 186 events ultimately included in the primary HRSD the median number of normal samples contributing to the event-specific reference was 112 (IQR = 103–113; range = 15–113). For each event, the median PSI in normal breast tissue was used as the reference value, and normal-tissue dispersion was estimated using the scaled median absolute deviation (1.4826 × MAD). When the MAD was unavailable or near zero, the standard deviation (SD) was used instead. Events with insufficient dispersion were excluded. The normal samples therefore provided a tissue-level reference for quantifying tumor-associated splicing deviation. Gene symbols were harmonized before intersection with the curated gene set, if necessary. Events satisfying QC were then intersected with the prespecified 73-gene HR set, yielding 199 AS events across 51 genes (Supplementary Table S1). Thirteen *POLH/POLK* events were excluded, leaving 186 AS events for subsequent analyses.

### 2.4. Construction of the Homologous Recombination Splicing Disruption Score (HRSD)

For each of the 186 QC-passing events, tumor-specific splicing disruption was quantified relative to normal breast tissue. The difference between each tumor PSI value and the corresponding normal-tissue median was divided by the event-specific normal-tissue dispersion estimate. The absolute standardized deviation was used to quantify the magnitude of splicing disruption irrespective of its direction. This normal-referenced standardization quantified the magnitude of deviation from the event-specific splicing pattern observed in normal breast tissue while accounting for normal-tissue variability.

A hierarchical scoring approach was used to prevent event-rich genes and larger HR modules from dominating the score. Within each tumor, event-level disruption values were summarized by the median within each gene, and gene-level values were subsequently summarized by the median within each of the eight HR functional modules. Gene and module scores required valid measurements for at least 70% of the events or genes contributing to each score, respectively. The eight module-level scores were then summarized by their median to generate a module-balanced HRSD. This aggregation provided balanced representation of the HR machinery by limiting the influence of genes represented by many AS events and modules containing larger numbers of genes. The primary HRSD was restricted to tumors with valid scores for all eight modules. Higher HRSD values indicated greater overall deviation of HR-associated splicing from the normal breast-tissue reference. HRSD values were standardized to z-scores for downstream analyses.

### 2.5. Homologous Recombination Deficiency (HRD) and Molecular Characterization

Genomic HRD was evaluated using the continuous HRD score described earlier. For categorical analyses, tumors with HRD scores ≥ 42 were classified as HRD-high based on the previously established threshold [40]. This binary classification was used as a secondary analysis, whereas genomic HRD score was analyzed as a continuous variable in the primary analyses.

Somatic mutation status was determined from non-silent variants. *BRCA1/2* somatic wild-type was defined as the absence of qualifying non-silent somatic mutations in either *BRCA1* or *BRCA2*. Additional HR-gene somatic mutation status was evaluated using a selected set of HR-related genes comprising *PALB2*, *RAD51C*, *RAD51D*, *BRIP1*, *BARD1*, *ATM*, *ATR*, *CHEK1*, *RAD50*, *MRE11A*, *NBN*, *RBBP8*, *FANCA*, and *FANCD2*. *TP53* somatic mutation status was defined using the same non-silent variant criteria.

*BRCA1* promoter methylation was assessed using four previously established promoter-associated CpG probes (cg19531713, cg19088651, cg08993267, and cg04658354) [41]. For each tumor, methylation β values were averaged across the four probes, and tumors with a mean β value ≥ 0.20 were classified as *BRCA1*-hypermethylated based on the previously established threshold [42,43]. PAM50 subtype annotations were used to distinguish basal from non-basal tumors for subtype-specific analyses.

### 2.6. Association of HR Splicing Disruption with Genomic HRD

The association between HRSD and genomic HRD scores was initially assessed using Spearman rank correlation (*ρ*). To evaluate whether this association persisted independently of established HRD-associated molecular features, multiple correlation analyses were performed in progressively restricted cohorts based on *BRCA1/2* somatic mutation status, selected additional HR-gene somatic mutations, *TP53* mutation status, and *BRCA1* promoter methylation. A strict mechanism-excluded cohort was defined as *BRCA1/2* somatic wild-type, *TP53* wild-type, *BRCA1*-unmethylated tumors without somatic mutations in the selected additional HR-genes. The analysis was also repeated after exclusion of basal tumors to assess whether the association was independent of the HRD enrichment characteristic of this molecular subtype.

Multivariable linear regression was used to evaluate the association between standardized HRSD and continuous HRD score, while multivariable logistic regression was used for HRD-high status. Both models were adjusted for *BRCA1/2* somatic mutation status, selected additional HR-gene somatic mutations, *TP53* mutation status, *BRCA1* promoter methylation, and PAM50 molecular subtype. Effects were reported per 1-SD increase in HRSD as regression coefficients with 95% confidence intervals (CI) for continuous HRD and odds ratios (OR) with 95% CI for HRD-high status.

### 2.7. HRD-Associated Splicing and Immunophenotypes

As an exploratory proof-of-concept analysis, we examined whether HRD-associated splicing features were associated with immune phenotypes derived from bulk tumor data. Six immunophenotypes representing complementary aspects of the tumor immune microenvironment were selected from established PanCancer immune measures: antigen presentation, interferon response, cytotoxic effector activity, checkpoint activity, regulatory T-cell abundance, and macrophage M1/M2 polarization [17,29]. The individual immune signature scores and CIBERSORT-derived cell fractions contributing to each phenotype were standardized before construction of the composite scores.

HRD-associated HR modules, genes, and individual AS events were evaluated for associations with the six immunophenotypes using Spearman rank correlation (*ρ*). Multiple-testing correction was performed using the Benjamini–Hochberg method, with a false discovery rate (FDR) < 0.05 considered statistically significant. Downstream analyses focused on regulatory T-cell and checkpoint/regulatory phenotypes, with the latter defined as the mean of their standardized scores. Multivariable linear regression was used to determine whether associations of prioritized HR modules, genes, and AS events with these immunoregulatory phenotypes persisted after adjustment for HRD score and PAM50 subtype, with standardized β coefficients and 95% CI reported (Supplementary Tables S2 and S3).

### 2.8. Characterization of RAD54B Alternative Termination

RAD54B|AT|84553 was prioritized based on its convergent associations with HRD and immunoregulatory phenotypes. Specifically, prioritization considered the strength of the HRD association, associations across multiple immune phenotypes, persistence of immunoregulatory associations after adjustment, and the biological interpretability of the reciprocal termination events. Alternative termination (AT) represents alternative transcript-ending events, distinguishing it from conventional internal splicing events. Event usage was evaluated in relation to HRD score, checkpoint/regulatory phenotypes, tumor versus normal tissue, and PAM50 molecular subtype. The differences between groups were assessed using Wilcoxon rank-sum or Kruskal–Wallis tests, as appropriate.

The reciprocal events, RAD54B|AT|84553 and RAD54B|AT|84556, were further examined for concordance and directional associations with HRD. PSI values were compared between HRD-high and HRD-low tumors in the overall cohort and after restriction to *BRCA1/2* somatic and wild-type tumors. Transcript and protein mapping was used to characterize the predicted coding consequences of alternative termination, including the retention of the *RAD54B* SWI2/SNF2 motor region (Supplementary Table S4). Finally, multivariable linear regression assessed associations between RAD54B|AT|84553 usage and the regulatory T-cell and checkpoint/regulatory phenotypes after adjustment for HRD score, PAM50 subtype, and *TP53* mutation status.

### 2.9. External Validation of RAD54B Alternative Termination

External validation was performed using the independent SCAN-B breast cancer cohort (GSE96058). *RAD54B* transcript models were reconstructed from the corresponding hg38 transcript annotation, and transcript expression was grouped according to terminal-exon structure to derive distal termination usage. Technical replicates were excluded, yielding 3273 independent tumors.

For structural validation, genomic coordinates of TCGA *RAD54B* alternative termination events were converted from hg19 to hg38 using the “GenomicRanges” [44] and “rtracklayer” [45] R packages and compared with *RAD54B* terminal-exon structures in SCAN-B. Biological validation was assessed by comparing distal termination usage across PAM50 subtypes using the Kruskal–Wallis test. Cross-cohort concordance was assessed by Spearman correlation of subtype-specific median RAD54B|AT|84553 PSI in TCGA-BRCA and distal termination usage in SCAN-B across the four major PAM50 subtypes.

### 2.10. Statistical Analysis

Statistical analyses were performed in the R environment (version 4.5.0) [46]. Continuous associations were assessed using Spearman rank correlation. Comparisons between two groups were performed using the Wilcoxon rank-sum test, while comparisons across multiple groups were performed using the Kruskal–Wallis test. Multivariable associations were evaluated using linear or logistic regression, as appropriate, with effect estimates reported with 95% confidence intervals. For analyses involving multiple comparisons, *p* values were adjusted using the Benjamini–Hochberg method, with false discovery rate (FDR) < 0.05 considered statistically significant. All statistical tests were two-sided, and unadjusted *p* < 0.05 was considered statistically significant where multiple-testing correction was not applicable. Data visualization was primarily performed using the “ggplot2” package in R [47].

## 3. Results

### 3.1. Construction of a Homologous Recombination Splicing Disruption Framework

An analytical framework was developed to evaluate whether alternative splicing (AS) of homologous recombination (HR) repair genes is associated with homologous recombination deficiency (HRD) and immune regulation in breast cancer (Figure 1). Analysis of a prespecified 73-gene HR set identified 199 QC-passing AS events across 51 HR genes in TCGA-BRCA, while 22 genes had no events meeting the QC criteria (Supplementary Table S1).

Of the 51 genes represented after event-level QC, 49 contributed to the primary HR splicing disruption score (HRSD) with 186 AS events (Supplementary Table S5). The genes were distributed across eight HR functional modules: *BRCA-PALB2* coordination, damage recognition/end resection, Fanconi HR interface, HR damage signaling, *RAD51* loading/strand invasion, recombination intermediate processing/resolution, repair DNA synthesis, and single-stranded DNA protection. The resulting HRSD provided a tumor-level representation of HR splicing disruption for subsequent assessment related to HRD and immune phenotypes (Figure 1).

### 3.2. HR Splicing Disruption Is Associated with Homologous Recombination Deficiency

**Figure 2:**
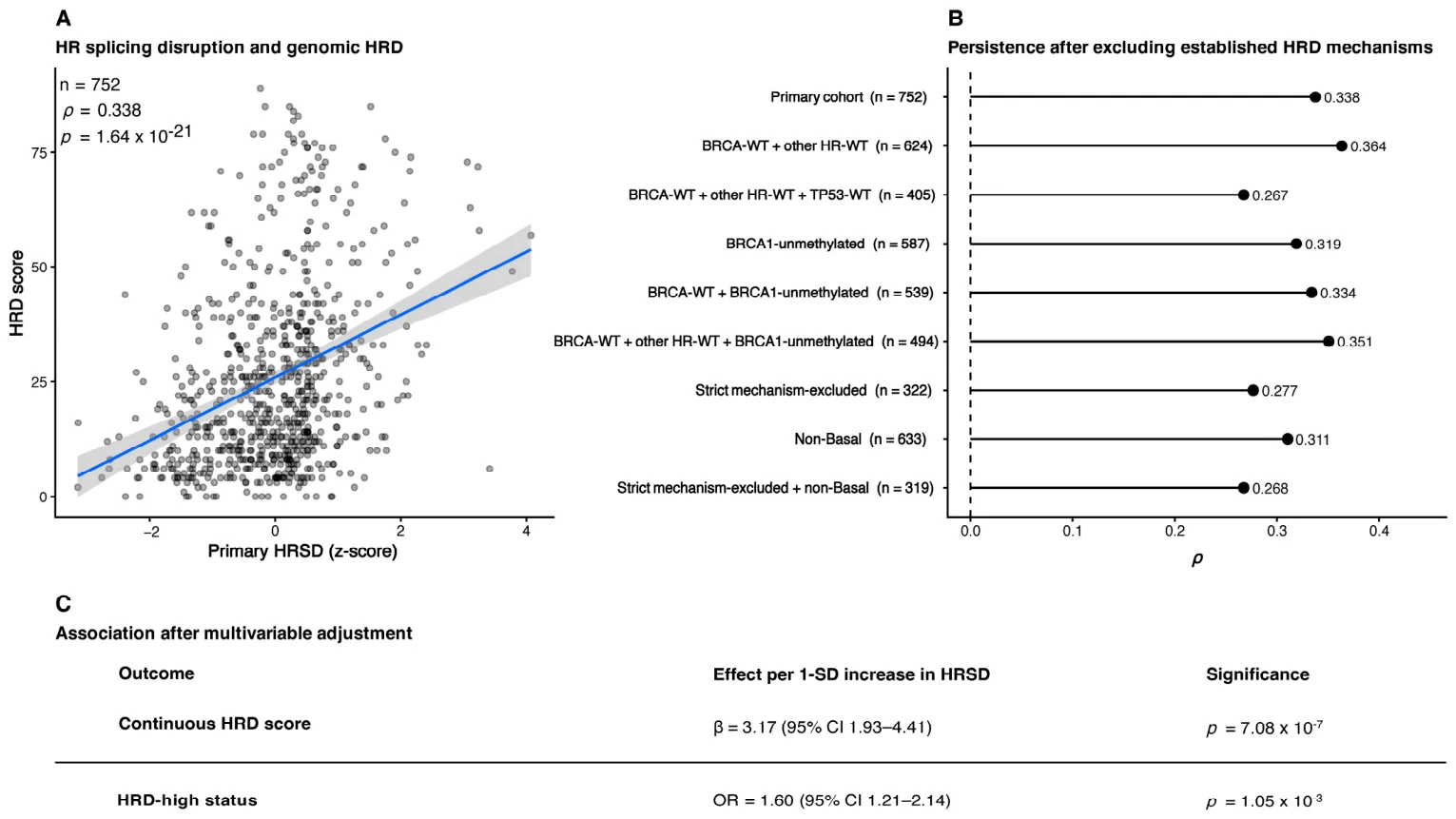
HR splicing disruption remains associated with genomic HRD after accounting for established HRD mechanisms. (**A**) Association between the homologous recombination splicing disruption (HRSD) score and genomic HRD score in the TCGA-BRCA cohort. Each point represents one tumor. The reported statistic is Spearman’s rank correlation along with the *p* value. The fitted linear trend and 95% confidence band are also shown. (**B**) Persistence of the HRSD-HRD association across progressively restricted molecular cohorts designed to reduce the contribution of established HRD-related mechanisms, including *BRCA1/2* somatic mutations, other selected HR-gene alterations, *TP53* mutation, *BRCA1* promoter methylation, and basal PAM50 subtype. Points indicate Spearman *ρ* values for each restricted cohort. (**C**) Multivariable models evaluating the association of HRSD with HRD after adjustment for relevant molecular covariates. HRSD remained associated with both HRD score and HRD-high status, with effects reported per 1-SD increase in HRSD along with their CIs and *p* values. OR, odds ratio; CI, confidence interval; PAM50, Prediction Analysis of Microarray 50.

HRSD was positively associated with HRD score in the primary TCGA-BRCA cohort (*ρ* = 0.338, *p* = 1.64 × 10^−21^; Figure 2A and Table 1). After excluding tumors with *BRCA1/2* somatic mutations and additional HR-gene alterations, the association was maintained (*ρ* = 0.364, *p* = 6.28 × 10^−21^). Moreover, the association remained significant after additional restriction to TP53 wild-type tumors (*ρ* = 0.267, *p* = 4.59 × 10^−8^).

HRSD also remained associated with HRD after accounting for *BRCA1* methylation (Figure 2B and Table 1). Positive associations were observed among *BRCA1*-unmethylated tumors (*ρ* = 0.319, *p* = 2.35 × 10^−15^), *BRCA1/2* somatic wild-type and *BRCA1*-unmethylated tumors (*ρ* = 0.334, *p* = 1.62 × 10^−15^), and after additionally excluding tumors with selected HR-gene alterations (*ρ* = 0.351, *p* = 9.63 × 10^−16^). In the strict mechanism-excluded cohort, restricted to *BRCA1/2* somatic wild-type, *TP53* wild-type, *BRCA1*-unmethylated tumors without the selected additional HR-gene alterations, the association remained significant *ρ* = 0.277, *p* = 4.49 × 10^−7^).

Given the well-established enrichment of HRD in basal-like breast cancers [48], we next assessed whether the association between HRSD and HRD persisted after excluding basal tumors. HRSD remained positively associated with HRD in the non-basal cohort (*ρ* = 0.311, *p* = 1.27 × 10^−15^) and in the strict mechanism-excluded, non-basal cohort (*ρ* = 0.268, *p* = 1.25 × 10^−6^) (Figure 2B and Table 1).

The association was further supported by multivariable analysis (Figure 2C, Table 1, and Supplementary Table S6). In the fully adjusted model, each 1-SD increase in HRSD was associated with a 3.17-unit increase in HRD score (95% CI, 1.93–4.41; *p* = 7.08 × 10^−7^) and higher odds of HRD-high status (OR = 1.6; 95% CI, 1.21–2.14; *p* = 0.001). These findings demonstrate a consistent association between HR splicing disruption and HRD across multiple molecular contexts.

### 3.3. HRD-Associated Splicing Features Converge with Immunoregulatory Phenotypes

**Figure 3:**
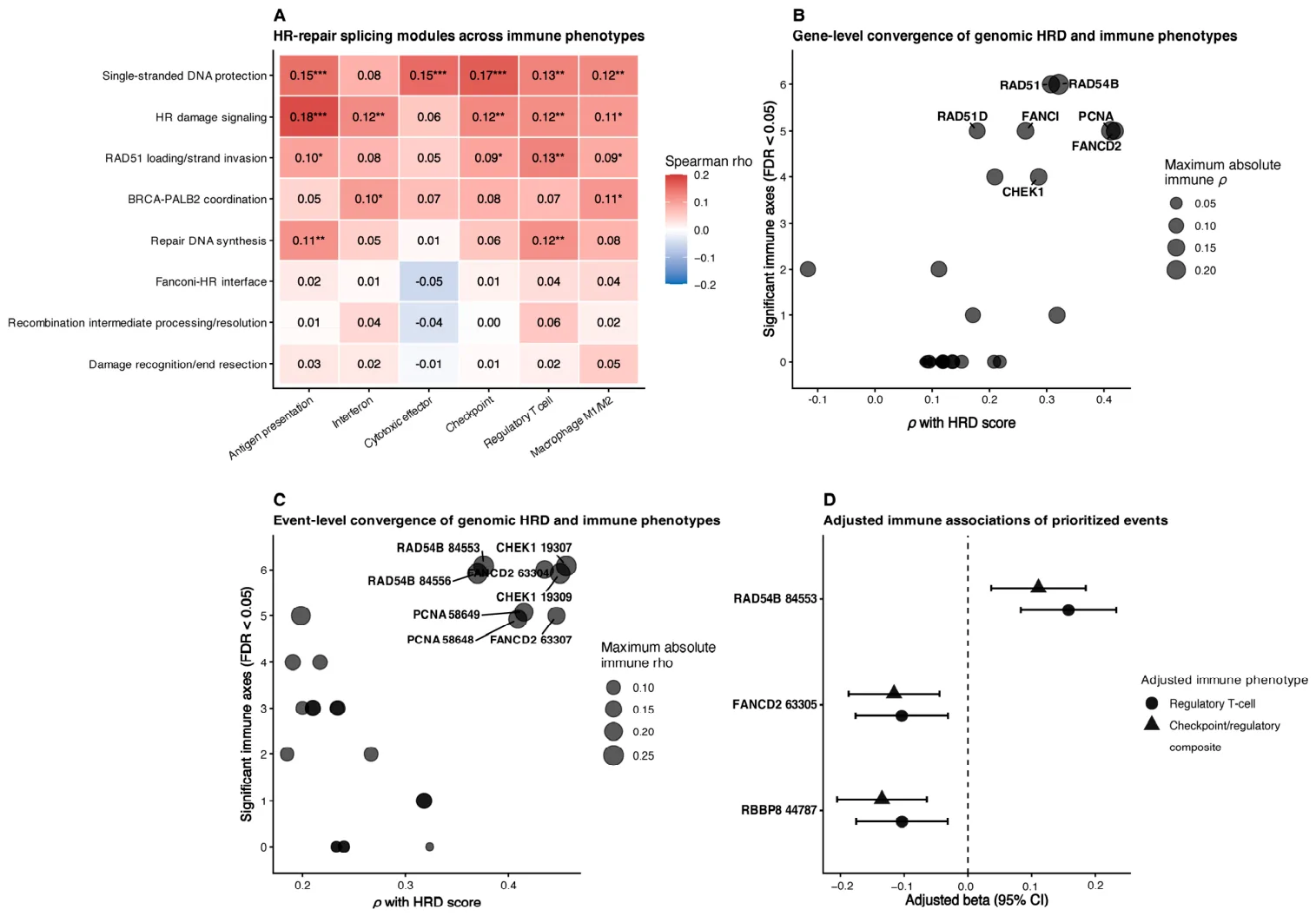
HRD-associated repair splicing converges with immune phenotypes. (**A**) Correlations between homologous recombination (HR)-repair splicing disruption at the module level (*y*-axis) and six immune phenotypes (*x*-axis). Cell values indicate Spearman correlation coefficients (*ρ*) with red and blue representing positive and negative correlations, respectively. Asterisks indicate statistical significance (* FDR < 0.05, ** FDR < 0.01, and *** FDR < 0.001). (**B**) Gene-level convergence of genomic homologous recombination deficiency (HRD) and immune phenotypes. The *x*-axis represents the correlation between gene-level splicing disruption and HRD score, whereas the *y*-axis indicates the number of immune phenotypes significantly associated with each gene after false discovery rate (FDR) correction. Bubble size represents the maximum absolute correlation with the evaluated immune phenotypes. (**C**) Event-level analysis of HRD-immune convergence using the same representation as in (**B**). Selected convergent events are labeled by gene and event identifier. (**D**) Adjusted associations of prioritized alternative splicing events with regulatory T-cell and checkpoint/regulatory phenotypes. Points indicate adjusted β coefficients and horizontal lines indicate 95% confidence intervals (CIs). The vertical dashed line indicates no association.

Given that HRD has been associated with remodeling of the tumor immune microenvironment [49], we next investigated whether HRD-associated splicing alterations also converged with immune phenotypes. HRD-associated splicing features showed associations with multiple immune phenotypes across module, gene, and event levels (Figure 3).

At the module level, five of the eight HR functional modules showed significant associations with at least one of the six evaluated immune phenotypes (Figure 3A and Supplementary Table S2). Single-stranded DNA protection and HR damage signaling showed the broadest immune associations, each being significantly associated with five immune phenotypes, while *RAD51* loading/strand invasion was associated with four. Both the *RAD51* loading/strand invasion and single-stranded DNA protection modules also showed significant associations with the checkpoint/regulatory composite and regulatory T-cell score that remained significant after adjustment for HRD and PAM50 subtype (Table 2 and Supplementary Table S2). For the checkpoint/regulatory composite, the adjusted associations were β = 0.09 (FDR = 0.0267) for *RAD51* loading/strand invasion and β = 0.135 (FDR = 0.0006) for single-stranded DNA protection. For the regulatory T-cell score, the corresponding associations were β = 0.096 (FDR = 0.028) and β = 0.125 (FDR = 0.002) for RAD51 loading/strand invasion and single-stranded DNA protection, respectively.

At the gene level, 25 HR genes were significantly associated with HRD and showed varying degrees of immune association (Figure 3B and Supplementary Table S2). *RAD54B* showed the broadest convergence, with significant associations across all six immune phenotypes. Its associations with the checkpoint/regulatory composite (β = 0.134, FDR = 0.00539) and regulatory T-cell score (β = 0.166, FDR = 1.57 × 10^−4^) remained significant after adjustment for HRD and PAM 50 subtype (Table 2 and Supplementary Table S2).

At the individual event level, multiple HRD-associated splicing events also showed associations across multiple immune phenotypes, with *RAD54B* alternative termination emerging as a prominent cross-domain feature (Figure 3C and Supplementary Table S3). The representative RAD54B|AT|84553 event retained significant associations with both the checkpoint/regulatory composite (β = 0.111, FDR = 0.0218) and the regulatory T-cell phenotype (β = 0.158, FDR = 4.85 × 10^−4^) after adjustment (Figure 3D and Table 2). Together, these findings support a consistent association between HRD-associated splicing alterations and immunoregulatory phenotypes across multiple levels of HR organization, with *RAD54B* emerging as a prominent cross-domain feature.

### 3.4. RAD54B Alternative Termination Is Associated with HRD and Immunoregulatory Phenotypes

**Figure 4:**
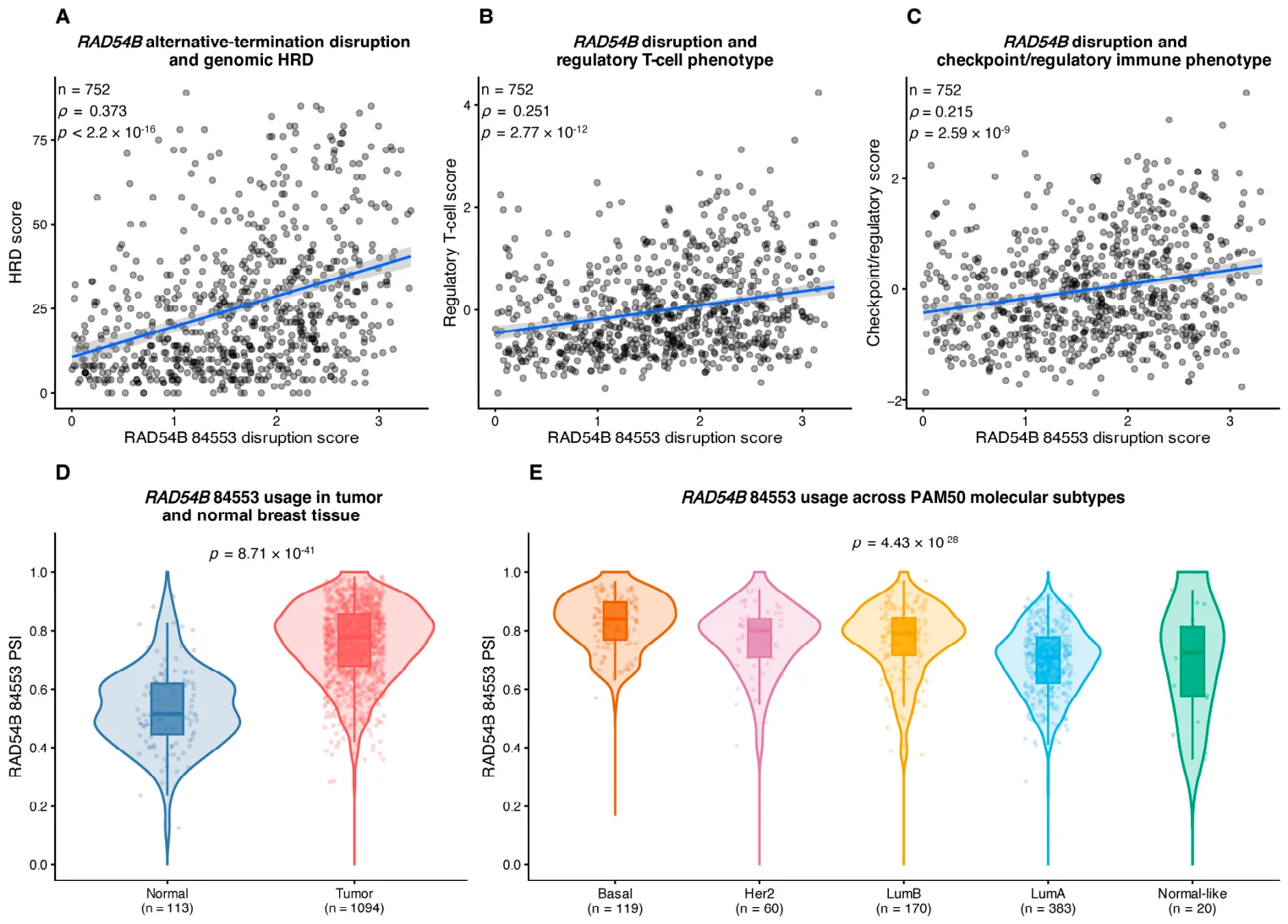
*RAD54B* alternative termination is associated with HRD and immunoregulatory phenotypes. Association between the RAD54B|AT|84553 disruption score and (**A**) homologous recombination deficiency (HRD) score, (**B**) regulatory T-cell score, and (**C**) checkpoint/regulatory score. Lines in panels (**A**–**C**) represent fitted linear trends with 95% confidence intervals (CIs). Correlation coefficients (*ρ*) along with their *p* values are shown on each plot. (**D**) Distribution of RAD54B|AT|84553 percent spliced in (PSI) values in normal breast tissue and primary breast tumors. Differences between groups were evaluated using the Wilcoxon rank-sum test. (**E**) Distribution of RAD54B|AT|84553 PSI across PAM50 molecular subtypes. Overall differences among subtypes were evaluated using the Kruskal–Wallis test. Sample sizes and corresponding statistical results are shown within the individual panels.

Given its cross-domain prioritization, we further characterized the representative RAD54B|AT|84553 alternative termination event. The event disruption was positively associated with HRD score (*ρ* = 0.373, *p* < 2.2 × 10^−16^; Figure 4A), with regulatory T-cell score (*ρ* = 0.251, *p* = 2.77 × 10^−12^; Figure 4B), and with checkpoint/regulatory phenotype (*ρ* = 0.215, *p* = 2.59 × 10^−9^; Figure 4C). RAD54B|AT|84553 usage was also markedly higher in breast tumors than normal breast tissue (median PSI 0.779 versus 0.516, *p* = 8.71 × 10^−41^; Figure 4D and Table 3) and differed significantly across PAM50 molecular subtypes (*p* = 4.43 × 10^−28^; Figure 4E), with highest median usage observed in basal tumors and the lowest in luminal A tumors. Together, these findings identified *RAD54B* alternative termination as an HRD-associated splicing feature with concurrent immunoregulatory, tumor-associated, and molecular subtype-specific patterns.

### 3.5. External Structural and Biological Validation of RAD54B Alternative Termination

**Figure 5:**
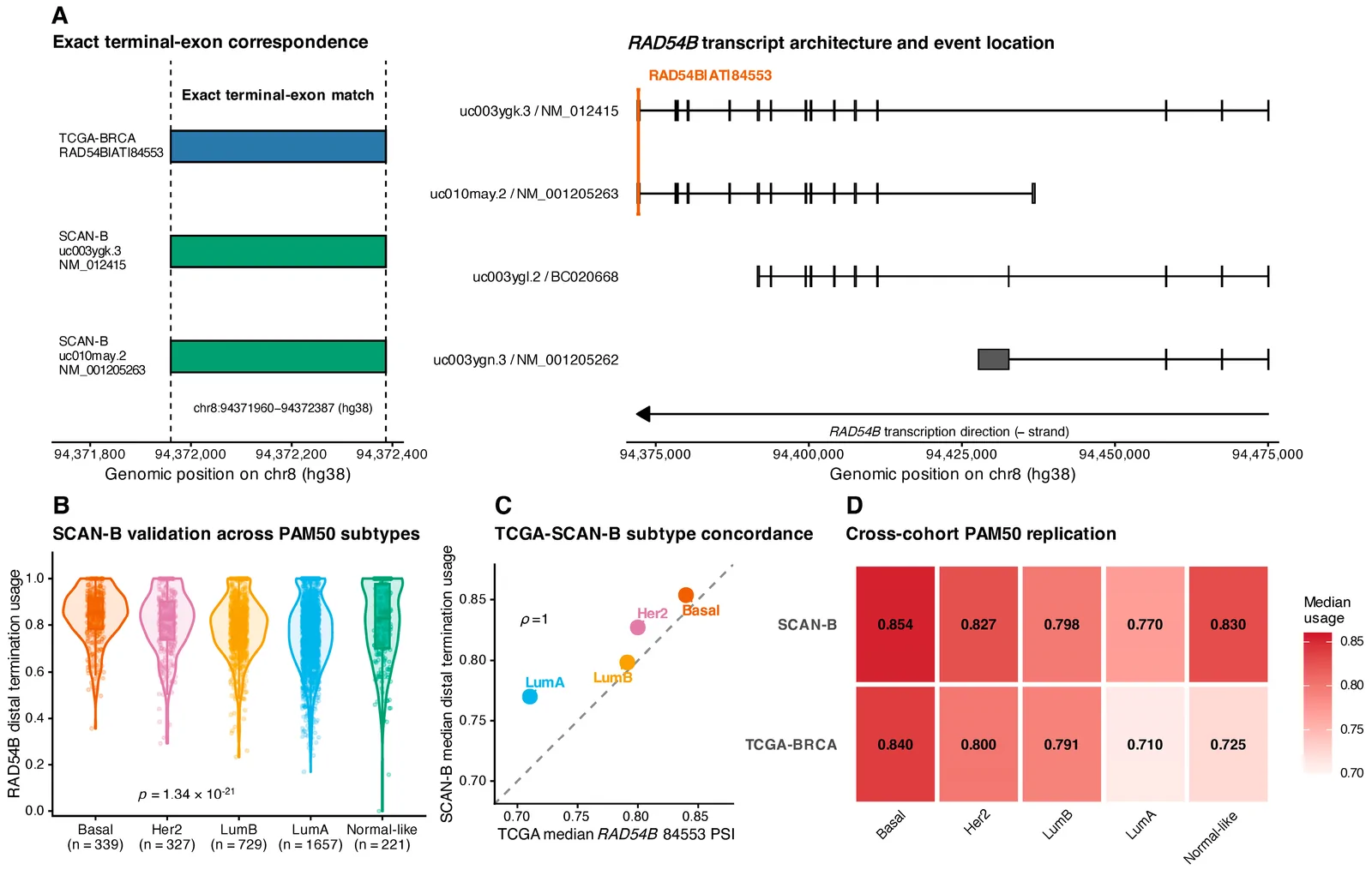
External structural and biological validation of *RAD54B* alternative termination in SCAN-B. (**A**) Structural correspondence between the TCGA-BRCA RAD54B|AT|84553 alternative termination event and *RAD54B* transcript models represented in SCAN-B. The left panel shows exact correspondence of the terminal exon associated with RAD54B|AT|84553 to the terminal exon of SCAN-B transcript models on chromosome 8. The right panel shows the genomic architecture of *RAD54B* transcript models and the location of the RAD54B|AT|84553 event (orange color). The arrow indicates the direction of *RAD54B* transcription on the negative strand. (**B**) Distribution of *RAD54B* distal termination usage across PAM50 molecular subtypes in SCAN-B. Differences among subtypes were evaluated using the Kruskal–Wallis test. Sample sizes and corresponding *p* value are shown. (**C**) Subtype-level concordance between median RAD54B|AT|84553 PSI in TCGA-BRCA and median distal termination usage in SCAN-B across the four major PAM50 subtypes. Correlation coefficient (*ρ*) is shown, and the dashed diagonal represents equal median usage between cohorts. (**D**) Cross-cohort comparison of median *RAD54B* termination usage across PAM50 subtypes in TCGA-BRCA and SCAN-B. Cell values indicate subtype-specific median usage, with color intensity representing the corresponding values. Normal-like tumors are shown for completeness but were not included in the correlation analysis in panel (**C**).

We next sought independent support for the *RAD54B* alternative termination phenotype using the SCAN-B cohort. Structural mapping demonstrated an exact correspondence between the terminal exon represented by RAD54B|AT|84553 in TCGA-BRCA and *RAD54B* transcript models identified in SCAN-B on chromosome 8 (Figure 5A and Table 3). This provided independent structural support for the alternative termination event identified in TCGA-BRCA.

*RAD54B* distal termination usage also differed significantly across PAM50 subtypes in SCAN-B (*p* = 1.34 × 10^−21^; Figure 5B and Table 3). The subtype-associated pattern was concordant between TCGA-BRCA and SCAN-B, with complete rank concordance across the four major tumor subtypes (*ρ* = 1; Figure 5C and Table 3). Consistent subtype-specific patterns of median termination usage were observed across the two cohorts (Figure 5D), providing independent structural and biological support for the *RAD54B* alternative termination phenotype.

### 3.6. Directional and Functional Characterization of RAD54B Alternative Termination

**Figure 6:**
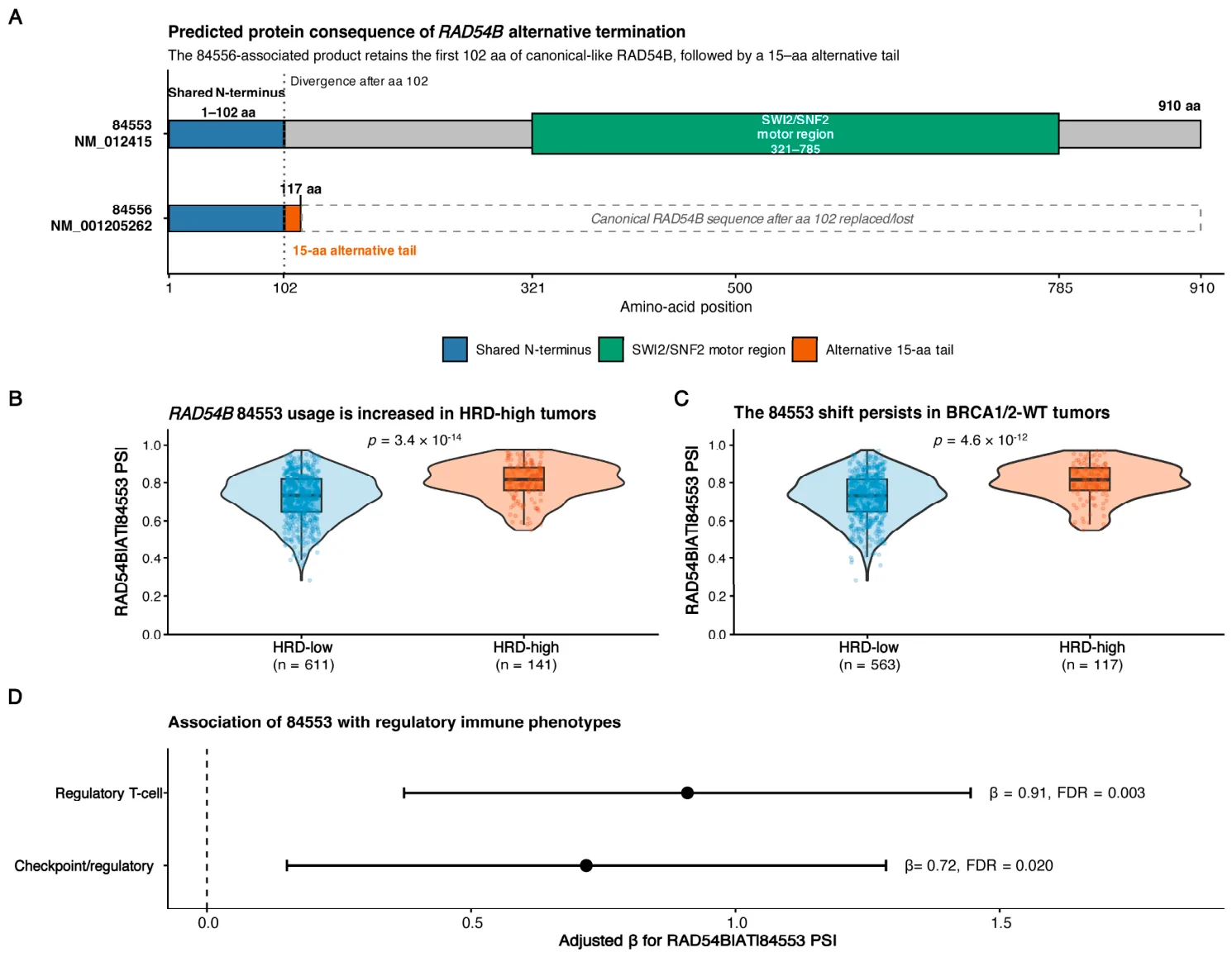
Protein-level consequences and molecular characterization of *RAD54B* alternative termination. (**A**) Predicted protein consequences of reciprocal *RAD54B* alternative termination. The 84553-associated transcript encodes a 910-amino-acid protein containing the SWI2/SNF2 motor region, whereas the 84556-associated transcript encodes a predicted 117-amino-acid product lacking this region. (**B**) Distribution of RAD54B|AT|84553 percent spliced in (PSI) values between HRD-low and HRD-high tumors. (**C**) The same comparison after restriction to *BRCA1/2* wild-type tumors. Differences between groups in panels (**B**,**C**) were evaluated using the Wilcoxon rank-sum test. Sample sizes and corresponding *p* values are shown. (**D**) Association between RAD54B|AT|84553 PSI and regulatory immune phenotypes. Points indicate adjusted coefficients (β) with 95% confidence intervals (CIs). The dashed vertical line indicates no association. Only associations with false discovery rate (FDR) < 0.05 are shown.

Further examination of the two *RAD54B* alternative termination events, RAD54B|AT|84553 and RAD54B|AT|84556, revealed a reciprocal relationship, with their PSI values summing to a median of 1.00 and showing a perfect inverse correlation (*ρ* = −1.00; Supplementary Table S4). Structural and protein mapping associated RAD54B|AT|84553 with the canonical-like 910-amino acid *RAD54B* product, whereas RAD54B|AT|84556 corresponded to a 117-amino acid product sharing the first 102 amino acids before divergence into a 15-amino acid alternative terminal sequence. The 84556-associated product was therefore predicted to lose 808 amino acids of the canonical sequence, including the SWI2/SNF2 motor region spanning amino acids 321–785 (Figure 6A and Supplementary Table S4).

The reciprocal termination pattern was directionally associated with HRD. RAD54B|AT|84553 usage was higher in HRD-high than HRD-low tumors (median PSI = 0.816 vs. 0.734, *p* = 3.42 × 10^−14^; Figure 6B and Table 3), whereas RAD54B|AT|84556 showed the reciprocal pattern (median PSI = 0.184 vs. 0.266, *p* = 3.42 × 10^−14^; Supplementary Table S4). Importantly, increased RAD54B|AT|84553 usage persisted in *BRCA1/2* somatic wild-type HRD-high tumors (median PSI = 0.814 vs. 0.733; *p* = 4.57 × 10^−12^; Figure 6C and Table 3).

Finally, RAD54B|AT|84553 usage remained independently associated with immunoregulatory phenotypes after adjustment for HRD score, PAM50 subtype, and *TP53* mutation status. Higher event usage was associated with the regulatory T-cell phenotype (β = 0.909; 95% CI, 0.373–1.44; FDR = 0.00274) and the checkpoint/regulatory phenotype (β = 0.718; 95% CI, 0.151–1.28; FDR = 0.0196) (Figure 6D and Table 3). Collectively, these findings support a directional association between RAD54B alternative termination and HRD, a predicted major alteration of *RAD54B* protein architecture, and an independent association with immunoregulatory phenotypes.

## 4. Discussion

In the current study, we investigated alternative splicing (AS) of the homologous recombination (HR) machinery as a transcript-level feature associated with homologous recombination deficiency (HRD) and the immune microenvironment in breast cancer. Using a normal-referenced, module-balanced framework, we identified a significant association between HR splicing disruption and genomic HRD that persisted across multiple molecularly restricted cohorts and after adjustment for established HRD-associated features. HRD-associated splicing also showed relationships with multiple immune phenotypes, with particularly consistent convergence involving checkpoint/regulatory and regulatory T-cell phenotypes. Cross-domain analysis further identified *RAD54B* alternative termination as a prominent feature associated with both HRD and immunoregulation. Detailed characterization demonstrated reciprocal *RAD54B* termination events with markedly different predicted protein products, while independent analysis in SCAN-B supported the corresponding terminal-exon structure and reproduced its molecular subtype-associated pattern. Together, these findings identify HR-associated AS as an additional transcript-level dimension of HRD biology in breast cancer.

The association between HR splicing disruption and genomic HRD extends previous evidence linking RNA processing with DNA-damage repair. AS is increasingly recognized as an important component of the DNA-damage response, and alterations in splicing can affect the processing and function of DNA-repair genes [50,51]. AS of major HR genes, including *BRCA1* and *BRCA2*, has been documented previously [14,15], while experimental perturbation of the splicing regulator *KHSRP* in breast cancer cells was recently shown to alter the splicing of multiple DNA-repair genes [16]. Our findings extend these observations from individual genes and experimentally perturbed systems to coordinated splicing variation across the HR machinery in primary breast tumors. Importantly, the normal-referenced HRSD was constructed hierarchically across events, genes, and functional modules, limiting disproportionate contributions from event-rich genes or larger HR modules. The observed association therefore reflects broader disruption across HR-associated splicing rather than variation in a single gene or event.

A particularly important finding was that the association between HRSD and genomic HRD persisted beyond several established HRD-associated molecular features. The relationship remained significant after sequential restriction according to *BRCA1/2* somatic mutations, selected additional HR-gene mutations, *TP53* mutations, and *BRCA1* promoter methylation. It also remained evident after exclusion of basal tumors, indicating that the association was not solely attributable to the enrichment of HRD within the basal-like subtype. HRD is known to arise through mechanisms beyond *BRCA1/2* mutations, including epigenetic inactivation of HR genes, with high-level *BRCA1* and *RAD51C* methylation associated with genomic HRD in breast and other solid tumors [6]. Conversely, comprehensive genomic analyses suggest that many HR-deficient tumors can ultimately be attributed to established genetic or epigenetic causes. The persistence of this association after accounting for several established HRD-related factors suggests that HR splicing disruption may provide additional transcript-level information about HRD. It remains unclear whether these splicing alterations contribute to HR impairment or arise in response to genomic stress.

The relationship between HRD-associated splicing and the immune microenvironment represents another important finding. Associations were observed across several of the six prespecified immune phenotypes, suggesting that HR-associated splicing relates to multiple aspects of tumor immunity. However, particularly consistent convergence was observed with checkpoint/regulatory activity and regulatory T-cell abundance. This pattern is compatible with previous evidence showing that HRD does not necessarily produce uniform effective anti-tumor immunity. In *BRCA1/2*-altered breast cancers, higher HRD scores have been associated with greater mutational and neoantigen burden, while also being associated with reduced cytolytic activity, immune infiltration, and type II interferon signaling [17]. Thus, the potential immunogenic consequences of genomic instability may coexist with mechanisms that limit effective immune responses.

Our findings extend this concept by showing that HRD-associated splicing also converges with immunoregulatory characteristics. Associations of the *RAD51* loading/strand invasion and single-stranded DNA protection modules with checkpoint/regulatory and regulatory T-cell phenotypes persisted after adjustment for HRD and PAM50 subtype, with similar findings observed for *RAD54B*. Given the established immunosuppressive role of regulatory T-cells in breast cancer [18], these findings suggest that HR-associated splicing may provide an additional transcript-level link between genomic instability and immune regulation.

Among the HR genes examined, *RAD54B* showed the broadest convergence between HRD and immune phenotypes, with RAD54B|AT|84553 emerging as a representative cross-domain event. This finding is biologically relevant because *RAD54B* is a SWI2/SNF2-family DNA-dependent ATPase involved in HR, and its inactivation has been shown to reduce homologous integration in human cells [52,53]. Detailed analysis revealed reciprocal RAD54B termination events with markedly different predicted products: RAD54B|AT|84553 corresponded to a canonical-like 910-amino acid product, whereas RAD54B|AT|84556 was associated with a predicted 117-amino acid product lacking most of the canonical sequence, including the SWI2/SNF2 motor region. Interestingly, HRD-high tumors showed increased usage of the canonical-like RAD54B|AT|84553 event rather than the predicted truncated event, suggesting that the termination shift may reflect altered transcript regulation associated with HRD rather than a simple loss-of-function mechanism. RAD54B|AT|84553 was also associated with regulatory T-cell and checkpoint/regulatory phenotypes after adjustment for HRD score, PAM50 subtype, and TP53 mutation status. To our knowledge, a relationship between *RAD54B* alternative termination and immune regulation has not previously been reported. Thus, *RAD54B* alternative termination represents a candidate transcript-level feature connecting HRD-associated splicing with immunoregulatory characteristics, although its functional significance requires experimental investigation.

Independent analysis of SCAN-B provided additional support for the *RAD54B* termination phenotype. Structural mapping demonstrated correspondence between the terminal exon represented by RAD54B|AT|84553 in TCGA-BRCA and *RAD54B* transcript structures reconstructed in SCAN-B. Moreover, distal termination usage differed across PAM50 subtypes and reproduced the subtype-associated pattern observed in TCGA-BRCA. These findings provide independent structural and biological support for the *RAD54B* termination phenotype. Further validation in cohorts with matched splicing, genomic HRD, and immune measurements would help establish the broader relevance of these associations.

The study provides new insights into the relationship between HR-associated AS, genomic HRD, and immune regulation in breast cancer. This may have broader implications for the molecular characterization of HRD. However, some limitations in the study should be considered. These include the retrospective nature of transcriptomic data, incomplete capture of all potential HRD mechanisms, potential residual confounding related to tumor purity and stromal/immune admixture in bulk tumor data, computationally derived immune phenotypes, and the need for experimental validation of the predicted *RAD54B* products. Nevertheless, our findings suggest that HR-associated splicing may provide complementary information about HRD and its immune context. Future studies integrating transcript isoform profiling with functional HR assays and treatment response data will be needed to determine the potential clinical relevance of HRSD and *RAD54B* alternative termination, including whether they provide incremental predictive information beyond established HRD biomarkers.

## 5. Conclusions

This study demonstrates that coordinated AS disruption across the HR machinery is associated with genomic HRD in breast cancer, including in tumors lacking several established HRD-associated molecular features. HRD-associated splicing also converged with immunoregulatory characteristics, particularly checkpoint/regulatory and regulatory T-cell phenotypes. *RAD54B* alternative termination emerged as a prominent feature linking these findings and showed independent structural and subtype-associated support in SCAN-B. Together, these findings identify HR-associated AS as an additional transcript-level dimension of HRD biology and support further investigation of its functional and clinical relevance.

## Figures and Tables

**Table 1:** Association of HR splicing disruption with HRD across multiple molecular cohorts.

Analysis	*N*	Effect Measure	Effect Estimate	*p* Value
Primary cohort	752	Spearman *ρ*	0.338	1.64 × 10^−21^
*BRCA1/2* somatic-WT; other selected HR mutations excluded	624		0.364	6.28 × 10^−21^
*BRCA1/2* somatic-WT; other selected HR mutations excluded; *TP53*-WT	405		0.267	4.59 × 10^−8^
*BRCA1*-unmethylated	587		0.319	2.35 × 10^−15^
*BRCA1/2* somatic-WT; *BRCA1*-unmethylated	539		0.334	1.62 × 10^−15^
*BRCA1/2* somatic-WT; other selected HR mutations excluded; *BRCA1*-unmethylated	494		0.351	9.63 × 10^−16^
Strict mechanism-excluded cohort *	322		0.277	4.49 × 10^−7^
Non-Basal PAM50	633		0.311	1.27 × 10^−15^
Strict mechanism-excluded cohort; non-Basal PAM50	319		0.268	1.25 × 10^−6^
Fully adjusted model: continuous HRD score	598	β per 1-SD HRSD (95% CI)	3.17 (1.93–4.41)	7.08 × 10^−7^
Fully adjusted model: HRD-high status	598	OR per 1-SD HRSD (95% CI)	1.60 (1.21–2.14)	0.001

* Strict mechanism-excluded cohort was restricted to BRCA1/2 somatic-WT, TP53-WT, BRCA1-unmethylated tumors without the selected additional HR-gene alterations. Abbreviations: HRSD, homologous recombination splicing disruption; HRD, homologous recombination deficiency; HR, homologous recombination; WT, wild-type; PAM50, Prediction Analysis of Microarray 50; SD, standard deviation; CI, confidence interval; OR, odds ratio.

**Table 2:** Cross-domain prioritization of homologous recombination splicing features associated with HRD and immune regulation.

Level	Feature	HRD *ρ* (FDR)	Checkpoint/Regulatory *ρ* (FDR)	Adjusted Checkpoint/Regulatory β (FDR)	Adjusted Regulatory T-Cell β (FDR)
Event	RAD54B|AT|84553	0.373 (7.08 × 10^−25^)	0.215 (1.62 × 10^−8^)	0.111 (0.0218)	0.158 (4.85 × 10^−4^)
Gene	*RAD54B*	0.321 (4.64 × 10^−18^)	0.216 (7.94 × 10^−8^)	0.134 (0.00539)	0.166 (1.57 × 10^−4^)
Module	*RAD51* loading/strand invasion	0.185 (8.57 × 10^−7^)	0.125 (0.002)	0.09 (0.0267)	0.096 (0.028)
Module	Single-stranded DNA protection	0.072 (0.05)	0.159 (9.56 × 10^−5^)	0.135 (0.0006)	0.125 (0.002)

Abbreviations: HRD, homologous recombination deficiency; FDR, false discovery rate; HR, homologous recombination.

**Table 3:** Characterization and cross-cohort validation of *RAD54B* alternative termination.

Dataset	Feature	Evidence	Result	Interpretation
TCGA-BRCA	RAD54B|AT|84553	HRD-high vs. HRD-low	0.816 vs. 0.734 (*p* = 3.42 × 10^−14^)	Increased usage in HRD-high tumors
TCGA-BRCA, BRCA1/2-WT	RAD54B|AT|84553	HRD-high vs. HRD-low	0.814 vs. 0.733 (*p* = 4.57 × 10^−12^)	Increased usage persists in *BRCA1/2*-WT
TCGA-BRCA	RAD54B|AT|84553	Adjusted regulatory T-cell association	β = 0.909 (FDR = 0.00274)	Positive independent association
TCGA-BRCA	RAD54B|AT|84553	Adjusted checkpoint/regulatory association	β = 0.718 (FDR = 0.0196)	Positive independent association
TCGA-BRCA	RAD54B|AT|84553	Tumor-normal PSI difference	0.779 vs. 0.516 (*p* = 8.71 × 10^−41^)	Increased usage in tumors
TCGA/SCAN-B	*RAD54B* distal termination	Structural validation	chr8:94371960–94372387 (exact match)	Corresponding structure identified in SCAN-B
SCAN-B	*RAD54B* distal termination usage	PAM50 subtype association	*p* = 1.34 × 10^−21^	Subtype-associated termination pattern
TCGA/SCAN-B	*RAD54B* distal termination usage	Cross-cohort PAM50 concordance	Spearman *ρ* = 1.00	Concordant subtype ranking across cohorts

Abbreviations: HRD, homologous recombination deficiency; PSI, percent spliced in; WT, wild-type; FDR, false discovery rate; CI, confidence interval; PAM50, Prediction Analysis of Microarray 50. Adjusted immune models included HRD score, PAM50 subtype, and TP53 mutation status. Cross-cohort PAM50 concordance was evaluated across the four major tumor subtypes (Basal, Her2, LumB, and LumA).

## Data Availability

All data supporting the findings of this study are provided within the article and its supplementary files. Alternative splicing profiles were downloaded directly from “TCGASpliceSeq” (https://bioinformatics.mdanderson.org/TCGASpliceSeq/, “accessed on 6 March 2026”). Genomic HRD data were downloaded as reformatted scores from the “TCGAhrd” repository (https://github.com/GerkeLab/TCGAhrd, “accessed on 12 March 2026”). PAM50 molecular subtype annotations and DNA methylation data were obtained from the NCI-GDC TCGA-BRCA 2012 publication resource (https://gdc.cancer.gov/about-data/publications/brca_2012, “accessed on 14 March 2026”. Immune-related data were obtained from the NCI-GDC PanCancer Atlas publication resource (https://gdc.cancer.gov/about-data/publications/panimmune, “accessed on 14 March 2026”.

## Supplementary Materials

The following supporting information can be downloaded at https://www.mdpi.com/article/10.3390/biomedicines14102231/s1. Table S1: Curated homologous-recombination gene universe used for HR splicing-disruption analysis; Table S2: Gene and module-level splicing features associated with HRD and immune regulation used for cross-domain prioritization; Table S3: Event-level HRD and immune associations used for cross-domain prioritization of homologous-recombination splicing events; Table S4: Detailed directional, structural, and functional characterization of RAD54B alternative termination; Supplementary Table S5. Alternative splicing events included in the primary HRSD score and their quality control characteristics, Supplementary Table S6. Univariable and multivariable associations of HRSD and established molecular features with genomic HRD score in TCGA-BRCA.

## Abbreviations

The following abbreviations are used in this manuscript:

AS	alternative splicing
AT	alternative terminator
CI	confidence intervals
DDR	DNA damage repair
FDR	false discovery rate
HR	homologous recombination
HRD	homologous recombination deficiency
HRSD	homologous recombination splicing disruption score
MAD	median absolute deviation
OR	odds ratios
PAM50	Prediction Analysis of Microarray 50
PSI	percent spliced in
SCAN-B	Sweden Cancerome Analysis Network-Breast
SD	standard deviation
TCGA-BRCA	The Cancer Genome Atlas Breast Invasive Carcinoma
